# Access to dental public services by disabled persons

**DOI:** 10.1186/s12903-015-0022-x

**Published:** 2015-03-13

**Authors:** Lyana Leal Rocha, Maria Vieira de Lima Saintrain, Anya Pimentel Gomes Fernandes Vieira-Meyer

**Affiliations:** Dentist of the Family Health Program - Secretaria Municipal de Saúde – Fortaleza, Ce, Rua do Rosário, 283, Fortaleza, Ce Brazil; Professor at the Public Health Master Program – UNIFOR, Av. Washington Soares, 1321 Sala S1, 60811-905 Fortaleza, Ce Brazil; Researcher at the Oswaldo Cruz Foundation – FIOCRUZ, Av. Santos Dumont, 5753 Sala, 1303, 60175-047 Fortaleza, Ce Brazil

**Keywords:** Disabled persons, Health services accessibility, Dentistry

## Abstract

**Background:**

According to the World Health Organization, one in every 10 people has a disability, and more than two-thirds of them do not receive any type of oral dental care. The Brazilian Constitution of 1988 guarantees all civilians including disabled people the right to healthcare, shaping the guidelines of the Brazilian National Health Care System (*Sistema Único de Saúde* – SUS). However, there is limited information about the true accessibility of dental services. This study evaluated the accessibility of public dental services to persons with disabilities in Fortaleza, Ceará, which has the third highest disability rate in Brazil.

**Methods:**

A cross-sectional quantitative study using structured questionnaires was administered to dentists (n = 89) and people with disabilities (n = 204) to evaluate the geographical, architectural, and organizational accessibility of health facilities, the communication between professionals and patients with disabilities, the demand for dental services, and factors influencing the use of dental services by people with motor, visual, and hearing impairments.

**Results:**

43.1% of people with disabilities do not recognize their service as a priority of Basic Health Units (BHU), 52.5% do not usually seek dental care, and of those who do (n = 97), 76.3% find it difficult to receive care and 84.5% only seek care on an emergency basis. Forty-five percent are unaware of the services offered in the BHU. Of the dentists, 56.2% reported difficulty in communicating with deaf patients, and 97.8% desired interpreters stationed in the BHU. People with disabilities gave better accessibility ratings than dentists (p = 0.001). 37.3% of the patients and 43.8% of dentists reported inadequate physical access infrastructure (including doors, hallways, waiting rooms, and offices). Dentists (60%) reported unsafe environments and transportation difficulties as geographical barriers, while most people with disabilities did not report noticing these barriers.

**Conclusions:**

While access to dental services has increased in Fortaleza, the lack of accessibility of health units and their surroundings does not promote the treatment of people with disabilities. Cultural, organizational, architectural, geographical, and communication barriers constrain the demand for and use of oral dental care services by this social segment.

## Background

More than one billion people in the world live with some form of disability. Despite the high prevalence of disability, a disproportionate percentage of disabled individuals do not receive oral dental care [1-3]. In Brazil, the Constitution of 1988 ensures the right to healthcare for all civilians. The constitution structures the guidelines of the Brazilian National Health Care System, also known as the Unified Health Care System (*Sistema Único de Saúde* – SUS) [4]. However, there is limited evidence of the accessibility of SUS oral health services to disabled persons.

The SUS organizes primary care through the Family Health Strategy (FHS), which is responsible for health promotion and the prevention, treatment, and rehabilitation of diseases and health conditions. The FHS team comprises a doctor, a nurse, a dentist, technical assistants, and community health agents [5]. The Oral Health Teams (OHT) consist of a dentist and FHS technical assistants.

The National Health Policy for Persons with Disabilities [6], supported by various national laws [7-9], ensures the rights of people with disabilities in many different fields, including dental care. OHTs must be able to embrace, assess, and provide dental care to all patients. Patients should only be referred to secondary care and tertiary (hospital) care when the capacity of the FHS service is exhausted [10,11]. FHS services, including OHTs, are provided in Family Health Care Units (FHU), which are located in neighborhoods and which form a strong link to the local community. Given their geographical and sociocultural proximity to the surrounding community, FHUs should be centers for excellence in care for people with disabilities [5,12]. However, information about the accessibility of Brazilian dental services in the literature is very limited.

The causes and consequences of disability vary from country to country, and consequences can differ depending on socioeconomic circumstances and state policies [13]. The lack of information on the identity, number, and geographical distribution of persons with disabilities, as well as on the nature of their disabilities, makes it difficult to design and implement targeted public healthcare policies in accordance with the actual needs of the population. In addition, little is known about the ability of the healthcare system to meet these needs. Fortaleza is the capital of the State of Ceará in northeast Brazil. It is the fifth largest city in the country, with approximately 2.5 million inhabitants. The Brazilian Institute of Geography and Statistics (*Instituto Brasileiro de Geografia e Estatistica* – IBGE) states that the State of Ceará has the third highest rate of disability (27.69%) in Brazil, higher than the northeastern (26.63%) and national (23.92%) rates [14]. Despite this, little is know about assess the accessibility of dental services by disabled persons in Fortaleza-Ceará.

The potential vulnerability of persons with disabilities is compounded because the incidence of caries and periodontal disease in people with special needs is higher than in the general population [3,15,16], and because these patients should be seen by dentists who understand their needs [17]. It is critical to provide this population access to public healthcare services that take into account their specific health, cultural, and social needs [6]. Thus, it is necessary to implement the National Policy for the Integration of Persons with Disabilities, ensuring equal access to oral health in the perspective of integrated care [18].

Accessibility is understood as the product of the relationship between the effective availability of health services due to the resistance presented by the environment, and the individuals’ access to these services [19]. It can also be understood as the ease with which persons with disabilities can use health services owing to the characteristics of the system and the services, and the chance they have to overcome organizational and geographical barriers [12] as well as barriers presented by the physical structure of the FHU and the difficulty of communication between professionals and patients.

This study aims to assess the accessibility of dental services in Fortaleza-Ceará, Brazil, to people with motor, visual, and hearing disabilities, considering the presence or absence of geographical, architectural, organizational, cultural, economic, and communication barriers.

## Methods

A quantitative cross-sectional study was conducted in the city of Fortaleza. Because of the lack of exact information about the number of people with disabilities in the city, the research estimated that 15% of the total population (2.5 million) had disabilities. This percentage was based on the percentage of people with disabilities in the region and access to local documents [14,20]. A probability sample of 204 people (the ‘disability’ group) with motor, hearing, or visual disabilities was obtained (sampling error of 7% and a 95% confidence interval). A sample of 89 dentists (the ‘dentist’ group) was recruited by randomly selecting one OHT dentist at each of the 89 FHU in the city. Data were collected using author-developed structured questionnaires, which were first piloted on 10 dentists and 10 persons with disabilities (not included in the samples) to adjust the instrument to account for the subjects’ perceptions, understanding, and ability to answer the questions.

The dentists were interviewed in the FHU where they worked to 1) assess geographical accessibility, the architectural structure of the FHU and dental offices, the communication between professionals and users with disabilities, and the organizational structure of dental services in the FHU; and 2) to identify the demand for dental services and factors that hinder or facilitate the use of these services by people with motor, visual, and hearing impairments.

The dentists and Community Health Agents (CHA) at each FHU helped to identify the persons with disabilities residing in the adjacent area. Interviews were carried out at individuals’ houses, at the Center for Integrated Medical Care (*Núcleo de Atenção Médica Integrada* – NAMI) of the University of Fortaleza (*Universidade de Fortaleza*), and at the Ceará Deaf Education Institute (*Instituto Cearense de Educação de Surdos* – ICES). NAMI was chosen because it is part of the SUS and is a reference center for the city of Fortaleza. The ICES is a bilingual school (Portuguese and Brazilian Sign Language – *Língua Brasileira de Sinais –* LIBRAS) and it is the only state public institution devoted exclusively to educational services for deaf people.

The results were analyzed using SPSS version 19.0 (SPSS Inc., Chicago, IL, USA). The Research Ethics Committee of the University of Fortaleza (*Universidade de Fortaleza* – UNIFOR) approved the study protocol (process No. 409/2011) and all participants signed an informed consent form.

## Results

Responses were collected from 293 people: 89 dentists and 204 people with a disability. The ages in the disability group ranged from 3 to 97 years (mean 39.8, SD = 22.7), while the dentists were aged between 25 and 55 years (mean 35.8, SD = 6.3).

In the disability group, 104 (51.0%) had a motor disability, 75 (36.8%) a hearing disability, and 25 (12.3%) a visual disability. One hundred and twelve (54.9%) were male, 71 (34.8%) had not completed high school, 62 (30.4%) dropped school before completing elementary school, and 24 (11.8%) had never attended school. One hundred and nine (53.4%) people were retired or pensioners, 109 (53.4%) were single, 195 (95.6%) lived with their parents or relatives, and 135 (66.2%) had a family income between one to two times the minimum wage (approximately US$330–660).

From the 204 individuals interviewed, 88 (43.1%) do not feel that their service is a priority. One hundred and seven (52.5%) reported not seeking dental services at the FHU on a regular basis, except in emergency situations (n = 82, 84.5%), while 23.5% (n = 48) used private services. Of those who seek care in the FHU (n = 97), 74 (76.3%) have difficulty receiving dental care. Forty five percent (n = 92) of users with disabilities do not know how dental services are organized in the FHU because they do not use them.

Of the dentists, 67 (75.3%) were women, 63 (70.8%) had completed a specialization program, 39 (43.8%) devoted their time exclusively to the FHS, and 60 (67.4%) had a family income of more than 10 minimum wages (approximately US$3,300). Their mean duration of work at the FHS was 7.9 years (SD = 3.3). A total of 74 (83.1%) dentists had no special training for working with disabled patients. Seventy-two dentists (80.9%) reported that there is no daily scheduling of dental visits for patients with disabilities. Forty-nine (55.1%) mentioned a long wait for consultation; 64 (71.9%) reported insufficient staff to meet the demand; 48 (53.9%) reported not feeling qualified to work with people with special needs because of the difficulty of the clinical management of these patients; and 72 (80.9%) reported difficulties in communicating with patients with disabilities, especially deaf people. In addition, 87 dentists (97.8%) reported the lack of LIBRAS interpreters in the FHU.

The architectural, geographical, organizational, and communicational characteristics of the FHU, from the point of view of dentists and people with disabilities, can be seen in Tables 1, 2, and 3. The tables show that dentists and people with disabilities evaluate these items differently.

**Table 1:** **Archtectural accessibility from the point of view of the persons with disability and the dentists**

**Archtectural accessibility**	**Persons with disability**		**Dentists**		p* value
	**n**	**%**	**n**	**%**	
**Adapted physical structure**					p < 0.001
Yes	94	46.1	19	21.3	
No	76	37.3	39	43.8	
Partially	-	-	31	34.8	
I do not know	34	16.6	-	-	
**Ramps**					p = 0.001
Yes	111	54.4	57	64.0	
No	65	31.9	32	36.0	
I do not know	28	13.7	-	-	
**Handrails**					p = 0.001
Yes	40	19.6	15	16.9	
No	136	66.7	74	83.1	
I do not know	28	13.7	-	-	
**Steps**					p < 0.001
Yes	56	27.5	44	49.4	
No	120	58.8	45	50.6	
I do not know	28	13.7	-	-	p = 0.001
**Parking spaces**					
Yes	8	3.9	7	7.9	
No	168	82.4	82	92.1	
I do not know	28	13.7	-	-	
**Wide doors**					p < 0.001
Yes	102	50.0	32	36.0	
No	74	36.3	57	64.0	
I do not know	28	13.7	-	-	
**Wide corridors**					p < 0.001
Yes	95	46.6	39	43.8	
No	81	39.7	50	56.2	
I do not know	28	13.7	-	-	
**Corridor signs**					p < 0.001
Yes	28	13.7	9	10.1	
No	148	72.6	80	89.9	
I do not know	28	13.7	-	-	
**Braille orientation in the FHC**					p = 0.001
Yes	3	1.5	1	1.1	
No	173	84.8	88	98.9	
I do not know	28	13.7	-	-	
**Adapted waiting rooms**					p < 0.001
Yes	107	52.5	29	32.6	
No	69	33.8	60	67.4	
I do not know	28	13.7	-	-	
**Adapted dental offices**					p < 0.001
Yes	109	53.4	33	37.1	
No	66	32.4	56	62.9	
I do not know	29	14.2	-	-	
**Adapted toilet**					p = 0.001
Yes	38	18.6	14	15.7	
No	138	67.6	75	84.3	
I do not know	28	13.7	-	-	
**Wheelchair**					p < 0.001
Yes	58	28.4	43	48.3	
No	118	57.8	46	51.7	
I do not know	28	13.7	-	-	

Fortaleza-CE, 2012.

Source: Developed by the author (2012). *Pearson’s chi-squared test.

**Table 2:** **Data on geographical accessibility from the point of view of the persons with disability and the Dentists - chi-squared test between variables**

**Geographical accessibility**	**Persons with disability**		**Dentists**		p* value
	**n**	**%**	**n**	**%**	
**Long time to get to the FHC**					p = 0.001
Yes	60	29.4	36	40.4	
No	114	55.9	53	59.6	
I do not know	30	14.7	-	-	
**Home very far from the FHC**					p = 0.001
Yes	44	21.6	25	28.1	
No	130	63.7	64	71.9	
I do not know	30	14.7	-	-	
**Needs a scort**					p < 0.001
Yes	146	71.6	86	96.6	
No	28	13.7	3	3.4	
I do not know	30	14.7	-	-	
**Dificulty with transportation**					p < 0.001
Yes	64	31.4	53	59.6	
No	110	53.9	36	40.4	
I do not know	30	14.7	-	-	
**High expenses with transportation**					p < 0.001
Yes	35	17.2	24	27.0	
No	139	68.1	65	73.0	
I do not know	30	14.7	-	-	
**Presence of slopes**					p < 0.001
Yes	24	11.8	34	38.2	
No	150	73.5	55	61.8	
I do not know	30	14.7	-	-	
**Presence of stairs**					p < 0.001
Yes	10	4.9	8	9.0	
No	164	80.4	81	91.0	
I do not know	30	14.7	-	-	
**Unsafe surroundings**					p < 0.001
Yes	48	23.5	58	65.2	
No	126	61.8	31	34.8	
I do not know	30	14.7	-	-	

Fortaleza-CE, 2012.

Source: Developed by the author (2012). *Pearson’s chi-squared test.

**Table 3:** **Data on organizational and communicational accessibility of dental services from the point of view of the person with disability and the Dentists**

**Organizational/communicational accessibility**	**Persons with disabilities**		**Dentists**		p* value
	**n**	**%**	**n**	**%**	
**Persons with disabilities receive priority care in the FHC**					p < 0.001
Yes	99	48.5	75	84.3	
No	88	43.1	6	6.7	
Partially	17	8.3	8	9.0	
**Type of priority**					p < 0.001
Home visit	19	9.3	-	-	
Examinations	1	0.5	-	-	
Scheduling of consultations	7	3.4	15	16.9	
Clinical care	31	15.2	53	59.5	
Do not wait in line	1	0.5	-	-	
Did not answer	145	71.1	21	23.4	
**Persons with disabilities seek dental services in the FHC**					p < 0.001
Yes	97	47.5	46	51.7	
No	107	52.5	43	48.3	
**FHC works during day and night time (up to 10 pm)**					p < 0,001
Yes	27	13.2	51	57.3	
No	85	41.7	38	42.7	
I do not know	92	45.1	-	-	
Dentist participate in the embracement**					
Yes	-	-	59	66.3	
No	-	-	30	33.7	
I do not know	-	-	-	-	
**Daily scheduling of dental consultations**					p < 0.001
Yes	25	12.3	17	19.1	
No	87	42.6	72	80.9	
I do not know	92	45.1	-	-	
**Existence of waiting list**					p < 0.001
Yes	72	35.3	27	30.3	
No	41	20.1	62	69.7	
I do not know	91	44.6	-	-	
**Long waiting time for care**					p < 0.001
Yes	73	35.8	49	55.1	
No	40	19.6	40	44.9	
I do not know	91	44.6	-	-	
**Referred through the reference system**					p < 0.001
Yes	106	52.0	81	91.0	
No	6	2.9	8	9.0	
I do not know	92	45.1	-	-	
**Return to the unit through the counter-reference system**					p < 0.001
Yes	97	47.5	67	75.3	
No	15	7.4	22	24.7	
I do not know	92	45.1	-	-	
**Enough Dentists to meet the demand**					p < 0.001
Yes	57	27.9	25	28.1	
No	55	27.0	64	71.9	
I do not know	92	45.1	-	-	
**Dentist qualified to work with persons with disabilities**					p < 0.001
Yes	83	40.7	41	46.8	
No	31	15.2	48	53.9	
I do not know	90	44.1	-	-	
Dentist can easily communicate with deaf people***					p < 0.001
Yes	58	77.3	50	56.2	
No	6	8.0	10	11.2	
Partially	6	8.0	29	32.6	
Never communicated	5	6.7	-	-	
**Libras interpreter in the FHC**					p < 0.001
Yes	4	2.0	2	2.2	
No	108	52.9	87	97.8	
I do not know	92	45.1	-	-	

Fortaleza-CE, 2012.

Source: Developed by the author (2012). *Pearson’s chi-squared test. **This question was not asked to persons with disabilities.

***This question was asked to people with hearing impairments and the Dentists.

## Discussion

To the authors’ best knowledge, this is the first study of this nature and scope to be performed in Brazil, with results that may reflect the national reality. Moreover, the state of Ceará ranks third in Brazil in the number of people with disabilities [20], which by itself justified the development of this study. This research contributes to the civil and scientific community, because the data obtained identify the characteristics of people with disabilities and the need for public policies targeting this population, and especially those with a low income and education level who lack access to existing goods and services.

The important findings include the lack of demand for general dental services by people with disabilities and, more specifically, the poor use of dental services provided by the SUS. Approximately one-third of people with disabilities said that they had never sought dental care in the FHU, and roughly 40% had never been examined by a dentist in Fortaleza’s public health service. The cause of this service gap is unclear, but it may be based on the sociodemographic, educational, and cultural profiles of people with disabilities, the lack of priority given to disability services, misunderstandings of the dental care system, and barriers to communication and access to healthcare services caused by geographical and architectural problems in the local area and in the FHU.

The prevalence of people with disabilities who were male and who had low income and education levels may explain the decreased demand for dental care and the poor use of services, given that sociodemographic factors, perceived need, beliefs, and the importance given to oral health strongly influence the use of dental services. These data are corroborated by Rohr and Barcellos [21] who found that women, people with a higher education level, and those with a better socioeconomic status are more likely to visit the dentist on a regular basis. Other studies show that men, and particularly those with lower levels of education, seek health services less frequently than women do because they see this type of care as a ‘woman’s thing’. Moreover, the opening hours of dental services match the men’s working hours, their needs are not met in a single consultation, and they tend to feel invulnerable to harm [22,23].

Although people with disabilities have achieved government policies that ensure their access to goods and services, health inequalities are still present [24]. They still experience difficulties in education and in the labor market because of their generally low level of education and lack of qualifications, and because of companies’ resistance to hire this group of people [18]. These difficulties affect other social sectors, including oral health, demonstrating the need to implement educational, healthcare, and rehabilitation interventions to reintegrate them into society.

The large percentage of this population that only seeks dental treatment for urgent care requires attention. Previous research corroborates the finding that pain is the main reason for seeking professional help [25]. The authors identified pain as a major reason for dental visits. They argued that most oral problems are not a threat to life and are, in general, treatable episodes, and that this perceived lack of urgency significantly limits the use of dental services [21]. Indeed, a large percentage of the users in our sample reported attending the FHU for dental emergencies only, rather than for preventive and restorative care.

This pattern of health-seeking behavior is not restricted to the city of Fortaleza or to Brazil. Research conducted in the province of Ontario, Canada, revealed that although 89% of dentists treat disabled people, only a small percentage of these people seek dental care, even though most of them recognize the importance of oral healthcare and have no barriers to physical access [26,27]. A large percentage of these individuals reported finding it very difficult to carry out their daily life activities and to access the dental facilities because over 70% require an escort. The specific difficulties in this sample can be explained, to some extent, by the greater proportion of people with motor disabilities and by the geographical and architectural barriers involved. Lack of autonomy is known to be an access-limiting factor, which may partially explain the low demand for oral services [28]. It is interesting to note that the ability of persons with disabilities to access health services autonomously depends, in addition to their physical condition, on mobility conditions in the streets and social spaces.

Notably, in Fortaleza, there was a difference in perception between dentists and persons with disabilities. A large number of dentists reported that they prioritized the care of people with disabilities in the basic healthcare unit, while a striking percentage of people with disabilities did not feel that their care was prioritized. Subjects with disabilities reported having difficulty receiving dental care at the basic healthcare unit in Fortaleza. Furthermore, although the SUS remains the preferred place of care for most of these subjects, 23.5% used private services. This finding is related to the subjects’ unfamiliarity with the organizational system of FHU dental services and may explain the low level of demand, justifying why a large number of people with disabilities do not use this service and therefore cannot answer questions on issues related to dental care.

The dentists’ biggest criticisms of geographical, architectural, and organizational barriers can be attributed to their higher educational and sociocultural level, when compared with people with disabilities. They have a different understanding of accessibility and a greater ability to problematize it. Their education leads dentists to understand that the real rights of people with disabilities are not being fully considered and will never be satisfied by partial improvements, while people with disabilities do not claim the fulfillment of these aspects.

The main organizational barriers identified by the dentists relate to the difficulty in booking dental appointments, despite the reception system for receiving the patient in the health unit and listening to their requests and needs. Dentists also cite the long waiting time for consultations and insufficient staff to meet demands, and more than half reported not feeling qualified to work with people with special needs because of the difficulty in the clinical management of and communication with these people, especially the deaf individuals. This need for training is also felt in other countries, with educational resources for dentists on providing care to special needs patients being considered essential to ensure the provision of high-quality care for those in need [17,29].

These barriers affect access, quality of care, and patient reception, thus compromising the outcomes of oral healthcare. The findings in Brazil are legitimated by other studies in which barriers related to the mode of health services organization stand out. Given the absence of protocols for scheduling, reception, and patient care within primary healthcare, accessibility is greatly influenced by the units’ organizational characteristics, which reflect the different local management and professional profiles [12,19].

Difficulties in communication are commonly emphasized as a barrier for patients with special needs [30]. The difficulty in communicating with the dentist during clinical care reported by the majority of deaf people and the lack of LIBRAS interpreters at the clinic necessitates the presence of a family member or a listener to intermediate the communication. This is corroborated by studies showing that the communication barriers, the lack of interpreters, and the lack of staff training specific to caring for this population are responsible for generating mismatch between legal decisions, the expectations of the deaf patients, and the actual services that can be offered at healthcare units [31].

According to the Brazilian Decree 5626/05 [32], health services must provide specific high-quality care for the deaf community to enable them to use dental services. In practice, this was not the reality found in Fortaleza’s FHU, where there were multiple barriers to adequate care for this community.

In keeping with Freitas et al. [33], who evaluated how deaf individuals perceived communication with dental professionals, this study observed that people with disabilities with low income are the ones who seek public healthcare services. These patients report that they are well assisted when they need dental care. However, the dentists do not know LIBRAS and they believe that the presence of interpreters in the FHU would facilitate communication with and care of those individuals with hearing loss.

Other studies have found that dentists try to maintain communication through written or verbal language, but because their speech is fast, lip reading is compromised, indicating that training dental professionals on the use of LIBRAS could improve accessibility by facilitating communication with deaf people, reducing the gap identified [33,34].

Although most people with disabilities did not emphasize geographical and architectural barriers as the main factors preventing access, approximately one-third of these users reported substantial time and difficulty traveling to the FHU. Likewise, they did not emphasize the lack of physical adaptations of the facility to meet their needs, including the lack of handrails and the lack of adaptation of doors, corridors, waiting rooms, and dental offices.

The difficulties in traveling to the health services identified by most dentists, including time, transportation, escort needs, and patient security, compromise accessibility. These findings are in agreement with those of Silva Junior et al. [35] concerning healthcare professionals from a sanitary district in Salvador, Bahia, Northeastern Brazil, where some coverage areas were far away from the FHU; this distance was a key barrier to access. Geographical accessibility is known to be an important factor in the effective use of health services, and the correct spatial distribution of services and patients will promote the appropriate use of health services [36].

Around 10% of dentists mentioned the poor condition of sidewalks and street paving. This observation is important because these conditions can impede or even prevent the mobility of people with disabilities, especially visually impaired individuals and people in wheelchairs. Vasconcelos and Pagliuca [37] mapped public roads in a city in Ceará, and they highlighted the unsafe conditions to which people with disabilities are exposed to on the way to healthcare facilities. They emphasized that these conditions limit free movement, especially for people in wheelchairs, who use crutches, or who have otherwise reduced mobility. In addition, the roads lacked audible traffic signals, tactile paving, and other adaptations that would facilitate the movement of people with disabilities.

The dentists identified barriers present in the physical structure of the FHUs that make them less suitable for people with special needs. They note that most FHUs in Fortaleza have no handrails and no parking spaces reserved for people with special needs. Most FHU corridors have no guide signs or information in Braille, and more than half have no adapted toilets or wheelchairs. The width of doors and corridors and the size of waiting rooms and dental offices were also identified as restrictions, corroborating other studies that show breaches of accessibility rules for healthcare units and the need to improve the physical infrastructure [28,37].

In this perspective, geographical accessibility describes the distance traveled by users to obtain healthcare, encompassing the linear distance, traveling time, and costs related to traveling [19,38]. Architectural accessibility includes the physical structure of the property insofar as it facilitates or impedes the easy access and movement of people with disabilities, preventing them from performing the most basic right of any citizen: to move freely [28].

Despite the numerous barriers identified in this study, there is evidence that access to dental services by the Brazilian population is improving. Comparing the data from the 1998 National Survey of Households (*Pesquisa Nacional por Amostra de Domicílios* – PNAD) to the data from PNAD/2008, the number of Brazilians who have never been to the dentist dropped from 19% to 7.8% [14].

The dentists in this study identified positive factors facilitating access to dental care, including that most units work during the third shift (night-time), dentists participate in patient reception, referrals are made to dental reference services for specialized care (*Centro de Especialidades Odontológicas* – CEO), and patients referred to CEOs are guaranteed return to basic healthcare units (counter-reference protocol).

These positive factors accord with the criteria outlined in the National Health Policy for Persons with Disabilities [39]. They show improvements in the processes of reception, care, reference, and counter-reference that are specialized to provide people with disabilities with better access to health facilities. Despite the many barriers to access found, the results of this study also demonstrate advances in the care of people with disabilities.

The institutionalization of more articulated and clearer practices concerning routines for the care and referral of patients in the public health system can positively affect accessibility [12]. However, patients should be made aware of this information through the visible disclosure of actions promoted by the health system; this communication is probably not reaching a population of individuals who do not exert their right as citizens.

Access to public dental services has recently expanded as a result of the implementation of the OHT in the FHS, the reorganization of the care model in primary care, and a greater supply and availability of health services. This study’s findings highlight the urgent need for actions aiming to improve access to dental services to promote inclusion and enable comprehensive care for people with disabilities. This process needs to be implemented worldwide. It is important to understand that a series of requirements need to be meet to better assist this population, including reducing social, economic, architectural, and geographical barriers. This article highlights some of the ways that these barriers influence access to dental care, both in Fortaleza and in situations found around the world. A limitation of this study is that only patients with certain specific physical disabilities were evaluated. Further investigation of other forms of disabilities is needed.

## Conclusion

Access to FHUs in Fortaleza for persons with motor, visual, and hearing disabilities is inadequate. Physical barriers include geographical location, travel time and cost, and lack of adaptive instruments including handrails, parking spaces, signage, adapted toilets, and wheelchairs. The low demand for and use of dental services at FHUs by persons with disabilities also results from cultural barriers, low awareness of SUS services in this population, and the urgent need to restructure services and processes including the scheduling of dental visits, patient reception, waiting time for consultations, and training of professionals. Communication barriers must be acknowledged and overcome to promote access for people with disabilities, both for emergency dental care and for preventive and restorative care.

## Abbreviations

CEO: Dental Reference Services for Specialized Care - *Centro de Especialidades Odontológicas*
CHA: Community Health Agents
FHS: Family Health Strategy
FHU: Family Health Care Unit
IBGE: Brazilian Institute of Geography and Statistics - *Instituto Brasileiro de Geografia e Estatistica*
ICES: Ceará Deaf Education Institute - *Instituto Cearense de Educação de Surdos*
LIBRAS: Brazilian Sign Language - *Língua Brasileira de Sinais*
NAMI: Center for Integrated Medical Care - *Núcleo de Atenção Médica Integrada*
OHT: Oral Health Teams
PNAD: National Survey of Households **-***Pesquisa Nacional por Amostra de Domicílios*
SPSS: Statistical Package for Social Sciences for Windows
SUS: Unified Health Care System - *Sistema Único de Saúde*
UNIFOR: University of Fortaleza - *Universidade de Fortaleza*
